# Removal of the Martin H. Smith Company

**Published:** 1902-06

**Authors:** 

**Affiliations:** New York


### CORRESPONDENCE.

### Removal of the Martin H. Smith Company.

SUCCESS AND GRATITUDE.

*Editor Buffalo Medical Journal :*

*Sir.*—We beg to gratefully announce to the medical profession the removal of our offices and laboratories to 105 Chambers Street, New York, where with greater space and more extensive facilities, we shall be better enabled to take care of the increasing demand for ergoapiol (Smith) and glyco-heroin (Smith).

After having our personal statements as to the unusual efficiency of these preparations in their respective indications, verified by hospital and clinical investigations, we sought to interest the physician individually, and the satisfaction of those who have investigated either or both could not be more fully manifested than in the necessity for our new and more commodious quarters.

Indeed, none the less is our gratitude to the BUFFALO MEDICAL JOURNAL, which has been so frequently mentioned in the communications requesting trial samples, etc., therein showing the large and wide-spread circulation of so consummate and estimable a journal.

MARTIN H. SMITH CO.

NEW YORK, May 25, 1902.

